# Editorial for the Special Issue on Fabrication, Characterization and Application of Organic/Inorganic Film Membranes and Advanced Materials (Volume III)

**DOI:** 10.3390/membranes16030107

**Published:** 2026-03-18

**Authors:** Elena Kalinina

**Affiliations:** 1 Laboratory of Complex Electrophysic Investigations, Institute of Electrophysics, Ural Branch of the Russia Academy of Sciences, 620016 Yekaterinburg, Russia; jelen456@yandex.ru; Tel.: +7-343-267-87-82; 2 Department of Physical and Inorganic Chemistry, Institute of Natural Sciences and Mathematics, Ural Federal University, 620002 Yekaterinburg, Russia

The Special Issue entitled “Fabrication, Characterization and Application of Organic/Inorganic Film Membranes and Advanced Materials (Volume III)” continues the themes of the previous Special Issue entitled “Fabrication, Characterization and Application of Organic/Inorganic Film Membranes and Advanced Materials (Volume II).” This issue contains nine articles covering current research areas, including proton-exchange membranes, hybrid polymer membranes, Li-conducting membranes, separation membranes for media separation devices, active chemical agents, oil fractions, membranes for solid oxide fuel cells, and biopolymer and layered membranes.

Qiang Bai et al. (Contributor 1) aimed to improve the performance of batteries based on open-cathode proton exchange membrane fuel cells (PEMFCs) and the characteristics of the membrane electrode assembly (MEA). The authors investigated the formation of the MEA unit and the effect of low-humidity operating conditions on cell performance. The capabilities of open-cathode PEMFCs have been previously demonstrated [1].

Nadezhda Tian et al. (Contributor 2) synthesized hybrid membranes with a poly(phenylene oxide) (PPO) matrix and 5 wt% copolyimide brushes with poly(methyl methacrylate) (PI-g-PMMA) for the separation of H_2_ + N_2_ and CO_2_ + N_2_ gas mixtures. The effect of saturating the gas mixtures with hexane, methanol, and water vapor on the permeability of the obtained membranes was studied. Diffusion coefficients for CO_2_ and N_2_ gases were calculated using Monte Carlo and molecular dynamics methods. The presented study is of great importance for solving the problems of natural gas purification from impurity vapors and increasing the selectivity of the separation membranes [2].

Mixed-matrix membranes (MMMs) can be successfully used to capture greenhouse gases, particularly CO_2_, making them environmentally significant [3]. Jiaming Gao et al. (Contributor 3) synthesized MMMs for CO_2_ capture in which the PIM-1 polymer matrix was modified with the amidoxime (UiO-66-AO) filler. The selectivity, permeability and resistance to aging of the obtained membranes were improved.

Li-ion batteries based on gel electrolytes with ionic liquid are a promising solution for increasing the stability, capacity, and safety of lithium-ion power sources [4]. Nikita A. Slesarenko et al. (Contributor 4) studied the properties of nanocomposite electrolyte membranes, which were obtained by the polymerization of polyethylene glycol diacrylate along with LiBF_4_, ethylene carbonate, and 1-ethyl-3-methylimidazolium tetrafluoroborate with the addition of TiO_2_ nanoparticles. Li-ion transport of the obtained membranes was studied using impedance spectroscopy, and analysis of the IR spectra and thermal analysis of the obtained material (DSC, TGA) were carried out. The obtained membranes were found to reach high conductivity at room temperature (4.8·10^−3^ S/cm), and can be used in Li-ion batteries with a LiFePO_4_ cathode and a gel electrolyte with ionic liquid. Experimental studies and quantum mechanical modeling revealed the features of lithium-ion transport in nanocomposite systems formed with the participation of nanoparticles.

Membrane technology can be successfully applied to the problem of separating crude oil fractions, in particular the removal of heavy fractions known as asphaltenes [5]. Aspects of this problem are reflected in the work of Alexey A. Yushkin et al. (Contributor 5), where the issues of synthesis and properties of membranes based on polyacrylonitrile (PAN), obtained by copolymerization with acrylic acid, were investigated. The proposed approach allowed the authors to reduce membrane fouling during ultrafiltration, which was demonstrated during the ultrafiltration of an oil/toluene mixture and the separation of asphaltene. The proposed solution is based on the use of polymer membranes formed from PAN; this polymer has high mechanical properties, resistance to solvents, low cost, stability to temperature effects, and chemical resistance [6]. Modification of the method of synthesis of polymer membranes using co-polymerization allowed the authors to increase their separating capacity.

Membrane technology allows users to solve the problems of separating media, active chemical agents, suspended particles, bacteria, and viruses [7]. It is important to ensure the resistance of membranes to solvents, in particular to the widely used organic solvent N,N-dimethylformamide (DMF). Ayang Zhou et al. (Contributor 6) developed methods for producing efficient separation membranes that are resistant to the DMF solvent. Membranes were obtained using liquefied walnut shells and modified with ethylenediamine (EDA). The obtained results demonstrated improved membrane permeability without degrading their separation properties. The separation properties of the membranes were demonstrated in various solvents, including DMF, dimethyl sulfoxide, ethanol, tetrahydrofuran, acetone, and ethyl acetate.

Dmitrii Agarkov et al. (Contributor 7) conducted a comprehensive study of the structural features, ionic conductivity, and phase composition of ceramic membranes based on ZrO_2_-Sc_2_O_3_ (ScSZ). Ceramic technology proved more advantageous for producing ScSZ ceramic membranes compared to crystalline ZrO_2_-Sc_2_O_3_ samples due to the pseudocubic structure of the solid solution formed during the manufacture of ceramic membranes. The technology for producing ScSZ ceramics included obtaining a coarse powder (30 μm) by grinding an ingot made by melting, followed by pressing, high-temperature sintering (1680 °C, 2 h), and cooling at a rate of 200 °C/h, which made it possible to preserve the cubic phase in the composition of the ceramics.

Yue Zhang et al. (Contributor 8) developed an approach to creating layered membranes with moisture resistance and antimicrobial activity. The membranes contained layers of Tencel/low-melting-point polyester-thermoplastic polyurethane/Triclosan (Tencel/LMPET–TPU/TCL). The obtained material can be used in medicine and for protective textiles. The morphological properties of the laminated membranes, including their water resistance, mechanical properties, and windproofness, were studied. The feasibility of mass production of the obtained layered membranes was demonstrated.

The development of biopolymer proton exchange membranes is of significant interest for the creation of microbial fuel cells [8]. Gowthami Palanisamy et al. (Contributor 9) developed a new biopolymer composite membrane material based on cellulose acetate (CA) containing SiO_2_ nanoparticles. The properties of the composite membrane were controlled by adding the plasticizer glycerol (G). The obtained CAG–2% SiO_2_ membrane had the high proton conductivity of 6.4 mS/cm. The addition of SiO_2_ particles improved the mechanical properties of the proton-exchange membranes.

The papers published in this Special Issue reveal the specifics of the synthesis of functional membrane materials in relation to their properties for application in promising areas of modern membrane technology, including applications in energy, chemical industry, medicine, and environmental solutions. I would like to express my gratitude to the authors of the articles in this Special Issue (Volume II and Volume III) for their insightful work, which is of interest to a wide range of researchers. I would also like to thank the co-editor of this Special Issue (Volume III), Prof. Dr. Konstantinos Beltsios.

## References

[1] Lei J., Zhao S., Liu Y., Deng X., Song G., Peng H. (2023). Effect of cathode channel structure on the performance of open-cathode proton exchange membrane fuel cell in dry environments. Energy Rep..

[2] Baker R.W., Lokhandwala K. (2008). Natural Gas Processing with Membranes: An Overview. Ind. Eng. Chem. Res..

[3] Dechnik J., Gascon J., Doonan C.J., Janiak C., Sumby C.J. (2017). Mixed-Matrix-Membranen. Angew. Chem. Int. Ed..

[4] Chen N., Zhang H., Li L., Chen R., Guo S. (2018). Ionogel Electrolytes for High-Performance Lithium Batteries: A Review. Adv. Energy Mater..

[5] Ganeeva Y.M., Yusupova T.Y.N., Romanov G.V.E. (2011). Asphaltene nano-aggregates: Structure, phase transitions and effect on petroleum systems. Russ. Chem. Rev..

[6] Tran T.D., Mori S., Suzuki M. (2007). Plasma modification of polyacrylonitrile ultrafiltration membrane. Thin Solid Film..

[7] Wang Z., Luo X., Zhang J., Zhang F., Fang W., Jin J. (2023). Polymer membranes for organic solvent nanofiltration: Recent progress, challenges and perspectives. Adv. Membr..

[8] Liu S.-H., Lee K.-Y. (2021). Optimization preparation of composite membranes as proton exchange membrane for gaseous acetone fed microbial fuel cells. J. Power Sources.

